# Supplementary material for: Antagonism between killer yeast strains as an experimental model for biological nucleation dynamics
Source: eLife. 2021 Dec 6;10:e62932. doi: 10.7554/eLife.62932 (PMC8730724; doi:10.7554/eLife.62932)
Supplement: Transparent reporting form [file elife-62932-transrepform1.pdf]

1st Floor
24 Hills Road
Cambridge CB2 1JP, UK

P 01223 855340
W elifesciences.org
T @elife

eLife's transparent reporting form

We encourage authors to provide detailed information *within their submission* to facilitate the interpretation and replication of experiments. Authors can upload supporting documentation to indicate the use of appropriate reporting guidelines for health-related research (see EQUATOR Network), life science research (see the BioSharing Information Resource), or the ARRIVE guidelines for reporting work involving animal research. Where applicable, authors should refer to any relevant reporting standards documents in this form.

If you have any questions, please consult our Journal Policies and/or contact us: editorial@elifesciences.org.

Sample-size estimation

- You should state whether an appropriate sample size was computed when the study was being designed
- You should state the statistical method of sample size computation and any required assumptions
- If no explicit power analysis was used, you should describe how you decided what sample (replicate) size (number) to use

Please outline where this information can be found within the submission (e.g., sections or figure legends), or explain why this information doesn't apply to your submission:

We did not have a priori knowledge on the variation of the data or effect size. Sample size was determined based on trial experiments. Sample sizes are reported in the figure legends for the experiments with liquid cultures and spatial well-mixed communities, and in the Materials and Methods section for the experiments with spatially structured populations.

Replicates

- You should report how often each experiment was performed
- You should include a definition of biological versus technical replication
- The data obtained should be provided and sufficient information should be provided to indicate the number of independent biological and/or technical replicates
- If you encountered any outliers, you should describe how these were handled
- Criteria for exclusion/inclusion of data should be clearly stated
- High-throughput sequence data should be uploaded before submission, with a private link for reviewers provided (these are available from both GEO and ArrayExpress)

Please outline where this information can be found within the submission (e.g., sections or figure legends), or explain why this information doesn't apply to your submission:

Details on experimental replication are reported in the figure legends and in the Methods section. During the measurement of competition assays with the flow cytometer, some wells of the 96 well measurement plates were not measured properly due to the aspiration of bubbles from the robotic liquid handler that automatically sampled from the 96 well plates. Due to the temporal sensitivity of the assay, relative frequency data from those replicates could not be recovered, and thus we excluded those technical replicates from the analysis, as stated in the Methods section. One outlier was encountered in the experiments reported in Figure 5, and was included in the analysis. The outlier was investigated experimentally after the experiment and the details of this investigation are discussed in the Results section (Mutations in killer production and sensitivity alter the outcome of competitions) and in Figures 6 and its supplement.

Statistical reporting

- Statistical analysis methods should be described and justified
- Raw data should be presented in figures whenever informative to do so (typically when N per group is less than 10)
- For each experiment, you should identify the statistical tests used, exact values of N, definitions of center, methods of multiple test correction, and dispersion and precision measures (e.g., mean, median, SD, SEM, confidence intervals; and, for the major substantive results, a measure of effect size (e.g., Pearson's r, Cohen's d)
- Report exact p-values wherever possible alongside the summary statistics and 95% confidence intervals. These should be reported for all key questions and not only when the p-value is less than 0.05.

Please outline where this information can be found within the submission (e.g., sections or figure legends), or explain why this information doesn't apply to your submission:

We included error bars that represent the standard error of the mean, the standard deviation or the range of our data in our measurements and reported in each figure legend what the error bars represent. In all two-point comparisons, the difference between the means is much greater than the sum of their standard errors and data from different treatments have non-overlapping ranges. In time courses, the standard errors are typically small in comparison to the difference in the values at successive time points. The data corresponding to each figure are included as source data files. Details on parameter estimation are reported in the section "Parameter fitting" of the Methods.

(For large datasets, or papers with a very large number of statistical tests, you may upload a single table file with tests, Ns, etc., with reference to sections in the manuscript.)

Group allocation

- Indicate how samples were allocated into experimental groups (in the case of clinical studies, please specify allocation to treatment method); if randomization was used, please also state if restricted randomization was applied
- Indicate if masking was used during group allocation, data collection and/or data analysis

Please outline where this information can be found within the submission (e.g., sections or figure legends), or explain why this information doesn't apply to your submission:

Group allocation does not apply to our experiments. Spatial randomization of treatments in liquid cultures competition experiments and spatial experiments was performed by distributing different technical replicates to random positions on 96 well plates (liquid competition experiments) and to random positions on a regular lattice on agar plates (spatially structured experiments), both irrespective of the treatment each replicate belonged to (i.e., inducer concentration and/or relative frequency of the two strains), as described in the Methods section.

Additional data files ("source data")

- We encourage you to upload relevant additional data files, such as numerical data that are represented as a graph in a figure, or as a summary table
- Where provided, these should be in the most useful format, and they can be uploaded as "Source data" files linked to a main figure or table
- Include model definition files including the full list of parameters used
- Include code used for data analysis (e.g., R, MatLab)
- Avoid stating that data files are "available upon request"

Please indicate the figures or tables for which source data files have been provided:

Source data were included for each figure. Codes used for data analysis and numerical integration of the model were uploaded on GitHub at the URL: https://github.com/andreagiometto/Giometto_Nelson_Murray_2020
